# High-Order Fiber Mode Beam Parameter Optimization for Transport and Rotation of Single Cells

**DOI:** 10.3390/mi12020226

**Published:** 2021-02-23

**Authors:** Zihao Shan, Shunnan Yao, Enfan Zhang, Dun Pi, Wen Cao, Feng Lin, Zhen Cai, Xingkun Wu

**Affiliations:** 1 State Key Laboratory of Modern Optical Instrumentation, College of Optical Science and Engineering, Zhejiang University, Hangzhou 310027, China; 3120100761@zju.edu.cn (Z.S.); 21730072@zju.edu.cn (D.P.); 2 Multiple Myeloma Treatment Center & Bone Marrow Transplantation Center, The First Affiliated Hospital, College of Medicine, Zhejiang University, Hangzhou 310003, China; yaoshunnan@outlook.com (S.Y.); efzhang@zju.edu.cn (E.Z.); 21818085@zju.edu.cn (W.C.); caiz@zju.edu.cn (Z.C.); 3 College of Engineering, Nanyang Technological University, Singapore 637457, Singapore; ASFLIN@ntu.edu.sg

**Keywords:** optical manipulation, single cell, high-order fiber mode, multiple optical traps

## Abstract

Optical tweezers are becoming increasingly important in biomedical applications for the trapping, propelling, binding, and controlled rotation of biological particles. These capabilities enable applications such as cell surgery, microinjections, organelle extraction and modification, and preimplantation genetic diagnosis. In particular, optical fiber-based tweezers are compact, highly flexible, and can be readily integrated into lab-on-a-chip devices. Taking advantage of the beam structure inherent in high-order modes of propagation in optical fiber, LP_11_, LP_21_, and LP_31_ fiber modes can generate structured radial light fields with two or more concentrations in the cross-section of a beam, forming multiple traps for bioparticles with a single optical fiber. In this paper, we report the dynamic modeling and optimization of single cell manipulation with two to six optical traps formed by a single fiber, generated by either spatial light modulation (SLM) or slanted incidence in laser-fiber coupling. In particular, we focus on beam size optimization for arbitrary target cell sizes to enable trapped transport and controlled rotation of a single cell, using a point matching method (PMM) of the T-matrix to compute trapping forces and rotation torque. Finally, we validated these optimized beam sizes experimentally for the LP_21_ mode. This work provides a new understanding of optimal optical manipulation using high-order fiber modes at the single-cell level.

## 1. Introduction

Fiber-based optical tweezers (FOT) have recently found a wide range of applications in manipulation of micro- and nanoparticles, as well as biological cells and biomolecules. In contrast to conventional optical tweezers (COT) requiring bulky microscope objectives and high numerical apertures to focus a laser beam, FOT, and especially single fiber optical tweezers (SFTs), can be implemented in very low-cost miniaturized systems or even biochips such as optofluidic platforms or catheters [1,2]. SFTs can also be used in particle suspensions in various environments such as microfluidics in arbitrary directions and depths for in situ particle manipulation [3,4,5].

In order to achieve complex manipulations such as single cell rotations, essential for medical, genetic, and cellular biological applications such as nuclear transplantations [6,7], embryo microinjections [8,9], and polar body biopsies [10,11,12,13,14], tweezer beams containing two or more field concentrations are often needed. These patterned beams can be generated using specialized multicore fiber [2,15], but more recently techniques taking advantage of the intrinsic structure in high-order fiber propagation modes with multiple lobes of coherent fields have come into the forefront. These types of single-core fiber optical tweezers can utilize conventional optical communication fibers with no specialized components and consist of a high-order guided mode or a linearly polarized mode (LP mode), generated by either spatial light modulator (SLM) or slanted incidence in the laser-fiber coupling [16,17,18].

While these new SFTs have experimentally demonstrated promise in complex manipulation such as precisely controlled rotation of single cells [18,19] and flexible arrangement of different particles [20] using inexpensive components, an adequate model is currently lacking to optimally size a high-order laser beam to transport arbitrary biological particle sizes, especially to facilitate cell capture, transport, and rotation using the same beam setup. Generally, radial and axial trapping efficiencies are strongly associated with small beam size and focus, as a tightly focused spot gives rise to a high trapping force. However, for rotation of single cells, a structured light field must be spread around a cell to form a driving torque on the cell when the light field is rotated. Therefore, an optimal beam size needs to be determined as a trade-off between the two conflicting demands. Furthermore, as bioparticles are subject to thermal and photo damage, it is crucial to apply a suitable beam to accomplish stable manipulation tasks with the lowest flux of laser energy onto the biological samples [21]. Most previous works utilized a focused laser beam with beam strengths in the order of hundreds of milliwatts, where both thermal and photo effects on bioparticles can be significant. As such, an in-depth characterization of laser beams in SFTs using different high-order modes can serve as a reference method for optimizing beam parameters for the manipulation of biological particles.

The interaction between a bioparticle and laser beam is determined by the scattering of a beam on a particle to change momentum and angular momentum of the light field, which is influenced by many factors, including overlap of the light field with dielectric particles, the difference in permeability between the particles and surrounding liquid substrate, heterogeneity in the structure of the particles, light intensity and wavelength, etc. A powerful tool developed for modeling of optical tweezers accounting for these factors is the T-matrix method [22], in which both optical force and torque acting on trapped particles can be accurately modeled by the elastic scattering of monochromatic light computed using a full-wave approach. The T-matrix method uses a response matrix, also called the T-matrix, which describes the properties of the micro-sized scattering particle and links the scattered- to incident-wave field by a linear superposition, as represented by a finite series in terms of a discrete basis set of Helmholtz eigenfunctions. Because the T-matrix is calculated only once for a specific bioparticle and is independent of illumination methods, this approach allows rapid repeated calculations of forces and torques under various illumination conditions for particles at different positions or orientations within a trapped beam with multiple traps.

In this study, we examine the optimization process of beam parameters for manipulation of single cells illuminated by structured light fields formed with high-order LP modes of single core fiber. Utilizing T-matrix modeling, we analyzed trapping and torque efficiencies of high-order modes of LP_11_, LP_21_, and LP_31_, and obtained an optimal ratio of light beam size as a function of target bioparticle size. On the basis of a combination of two-dimensional force maps with rotation torques created by multiple traps within a high-order mode beam, both trapped transportation and rotation of single cells are investigated in terms of various illumination conditions in an effort. Theoretical calculations are compared with experimental measurements performed on transporting and rotating a single yeast cell using LP_21_ beam to illustrate the effectiveness of the proposed modeling approach.

## 2. Materials and Methods

Modeling of single cell manipulation including rotation and transport by a focused beam of high-order fiber modes is developed using T-matrix method. By emitting a wavelength shorter than the cutoff wavelength of single mode operation in a standard G.652 communication fiber (refractive index difference Δn/n ≈ 0.003), LP modes with mode orders higher than the fundamental mode can be selectively excited and propagated with a low loss for optical tweezer applications. The LP_lm_ mode, as a superposition of the HE_l + 1,m_ and EH_l − 1,m_ modes, has a centrosymmetric intensity distribution. By solving the Helmholtz equation with proper boundary conditions for optical fiber, we obtained the spatial distribution of electromagnetic field of the LP_lm_ mode (see Supplementary Materials S1) as follows:

(1) $$E_{FFx}\left(R,\Theta ,\Phi \right)\approx iE_{l}\frac{e^{-ikR}}{kR}{\left(kaV\right)}^{2}\cos\left(l\Phi \right)\frac{ka\sin\left(\Theta \right)J_{l+1}\left(ka\sin\Theta \right)-V\sqrt{1-b}\frac{J_{l+1}\left(V\sqrt{1-b}\right)}{J_{l}\left(V\sqrt{1-b}\right)}J_{l}\left(ka\sin\Theta \right)}{\left[V^{2}\left(1-b\right)-k^{2}a^{2}\sin^{2}\Theta \right]\left[V^{2}b+k^{2}a^{2}\sin^{2}\Theta \right]}$$

where *k* = 2π/λ, a is the diameter of fiber core, V=ka·NA (NA, numerical aperture of optical fiber) is normalized frequency, *J_l_* (*x*) are Bessel functions, and b is the normalized phase constant. We limit our investigations of high-order modes up to LP_31_ with six lobes in the light field distribution. Above this order, multiple lobes of the field become so dense that a ring-like structured light field is formed, which cannot facilitate rotational manipulation of bioparticles.

Apart for the purpose of rotational manipulation, manipulation functions including single cell capture and transport must be maintained satisfactorily. We performed trapping force calculations for different bioparticle locations with respect to the center of the laser beam as a function of beam size, beam modes, and direction of trapping forces. In the following model, we assume the target biological particle to be a homogeneous isotropic spheroid-shaped cell with short axis of 4.2 μm, which is the target size selected for experimental validation using yeast cells. Previous work has shown that trapping force-displacement calculations using the T-matrix method was in excellent agreement with experimental measurements [23]. To solve for the T-matrix associated with a dielectric spheroid-shaped bioparticle, we utilized a point matching method (PMM), suitable for nonspherical particles. In our implementation of the PMM T-matrix calculation, both incoming and scattered electric fields are expressed in terms of Hankel functions and spherical harmonics as follows:

(2) $$E_{in}=\sum_{n=1}^{\infty }\sum_{m=-n}^{n}a_{nm}^{\left(2\right)}M_{nm}^{\left(2\right)}\left(kr\right)+b_{nm}^{\left(2\right)}N_{nm}^{\left(2\right)}\left(kr\right),$$

(3) $$E_{out}=\sum_{n=1}^{\infty }\sum_{m=-n}^{n}p_{nm}M_{nm}^{\left(1\right)}\left(kr\right)+q_{nm}N_{nm}^{\left(1\right)}\left(kr\right),$$

(4) $$M_{nm}^{\left(1,2\right)}\left(kr\right)=N_{n}h_{n}^{\left(1,2\right)}\left(kr\right)C_{nm}\left(\theta ,\varphi \right),$$

(5) $$N_{nm}^{\left(1,2\right)}\left(kr\right)=\frac{h_{n}^{\left(1,2\right)}\left(kr\right)}{krN_{n}}P_{nm}\left(\theta ,\varphi \right)+N_{n}\left(h_{n-1}^{\left(1,2\right)}\left(kr\right)-\frac{nh_{n}^{\left(1,2\right)}\left(kr\right)}{kr}\right)B_{nm}\left(\theta ,\varphi \right),$$

where $h_{n}^{\left(1,2\right)}\left(kr\right)$ are spherical Hankel functions of the first and second kinds, $N_{n}={\left[n\left(n+1\right)\right]}^{-\frac{1}{2}}$ are normalization constants, $B_{nm}\left(\theta ,\varphi \right)=r\nabla Y_{n}^{m}\left(\theta ,\varphi \right)$,$\;C_{nm}\left(\theta ,\varphi \right)=\nabla \times \left(rY_{n}^{m}\left(\theta ,\varphi \right)\right)$, and $P_{nm}\left(\theta ,\varphi \right)=\hat{r}Y_{n}^{m}\left(\theta ,\varphi \right)$ are the vector spherical harmonics, and $Y_{n}^{m}\left(\theta ,\varphi \right)$ are normalized scalar spherical harmonics, wherein *θ* and *φ* are the co-latitude and the azimuth in polar spherical coordinates, respectively. The expansion is truncated at some finite N_max_, determined by scatter size r_0_ and wavelength ($N_{max}\approx kr_{0}+\sqrt[{3}]{kr_{0}}$), resulting in 2N_max_ (N_max_ + 2) coefficients for each of the field expansions. In our calculation wavelength was set at 650 nm, r_0_ = 6 μm, and the N_max_ was up to 70 as required by calculation accuracy and model convergence. The wavelength of 650 nm was selected for both a high-order mode generation in a standard G.652 optical fiber and observation of cells in visible range by blocking laser beam with a high-pass filter. The expansion coefficients *p_nm_* and *q_nm_* are linked to those of incoming field *a_nm_* and *b_nm_* by a T-matrix, which was solved for by applying Maxwell equations on the boundary surface of the scatter. For a scatter devoid of symmetry, centered at origin, its surface can be specified by a function of angle *r = r (θ,* *φ)*. For simplicity, a grid of 2N_max_ (N_max_ + 2) points with equal angular spacing in each of *θ* and *φ* directions was set up. On the scatter surface, continuity of tangential components of both E- and H-fields by Maxwell equations links outside fields to those inside the scatter, yielding 8N_max_(N_max_ + 2) independent equations to solve for elements of T-matrix column by column. Thereby, all field coefficients (*a_nm_, b_nm_, p_nm_*, and *q_nm_*) are obtained for a given scattering entity and both forces, and thus torque can be directly calculated by using these coefficients [24,25]. This point matching method has the advantage of being simple to implement within a general T-matrix package for spheroid yeast cells in this study.

Controlled rotation of a captured bioparticle can be realized by rotating a focused beam with an asymmetric cross sectional intensity distribution. This rotational torque results from the nonuniform scattering of incoming angular momentum of the light field by the target bioparticle. The optical torque *M_z_=τ_z_·P/ω*, with P being laser power, *ω* the optical frequency, and *τ_z_* torque efficiency, can be calculated based on the scattering property of the particle for both momentum and angular momentum [24] as follows:

(6) $$\tau_{z}=\overset{\infty }{\underset{n=1}{\sum }}\overset{n}{\underset{m=-n}{\sum }}m\left({\left|a_{nm}\right|}^{2}+{\left|b_{nm}\right|}^{2}-{\left|p_{nm}\right|}^{2}-{\left|q_{nm}\right|}^{2}\right)/P$$

We performed experimental measurement of optical torque and trapping efficiency using a focused LP_21_ mode, which was generated by the slanted incidence of the laser into a fiber and focused by a fiber-tipped axicon lens with a conical angle of 135° [19]. Yeast cells suspended in DI water were used as samples for bioparticles, with its eccentricity ranging from 1.25 to 1.39, laser power P = 10 mW, and beam size 2*ω_0_* = 4.8 μm. Living cells in liquid substrate were pumped into a microfluidics system mounted on a three-dimensional (3D) piezoelectric nano-stage with a movement resolution of 20 nm (Max311D NanoMax Thorlabs, Thorlabs, Inc., Newton, NJ, USA). A CMOS camera (300E, SunTime Tech, Taiwan, China) recorded the image by a microscope system with a long working distance plan apochromat objective (GCO-2133, 40X, NA = 0.60, Daheng Optics, Beijing, China). The waist of the beam from the conical fiber was positioned close to the nozzle of microchannel of the microfluidics for selectively manipulation of passing-through cells. Single cells were illuminated by light-emitting diode (LED) white light. The light beam coming from LEDs was first collected and collimated by a set of Fresnel lenses, and then focused to the region under study in the microfluidic system through a microscope condenser. A tiny hole of 300 μm in diameter was drilled through the lenses of the condenser in the center. The optical fiber for optical trap, jacketed with a stainless sleeve, was placed through the hole to reach cells in microchannel. A high-performance short-pass filter (650 nm short-pass filter, FESH0650, Thorlabs, Newton, NJ, USA) was used to block the scattered laser light, and image exposure was adjusted by a variable neutral density filter (ND2-ND400) for an optimal contrast. The beam emanating from the fiber end was focused by an axicon lens micromachined at the exit end of the fiber with an adjustable beam waist diameter ranging from 3 to 7 μm, depending on the selected apex angle of the axicon lens. The captured single cell was levitated inside the microchannel, which was moved laterally by the piezoelectric nano-stage to produce a cell translation from beam center, using Stokes resistance for measurement of trapping efficiency at different field positions. Rotation of the single cell using the same beam was implemented by rotating structured light field by twisting a segment of the optical fiber propagating LP_21_ mode [17]. Sequences of frames recording cell transport and rotation manipulations were used to calculate Stokes viscous forces, torques, and peak torque during the rotation when cell sliding occurred in the process of rotation [19].

## 3. Results and Discussion

### 3.1. Landscape of Forces Generated by LP_11_, LP_21_, and LP_31_ Modes

**Figure 1:**
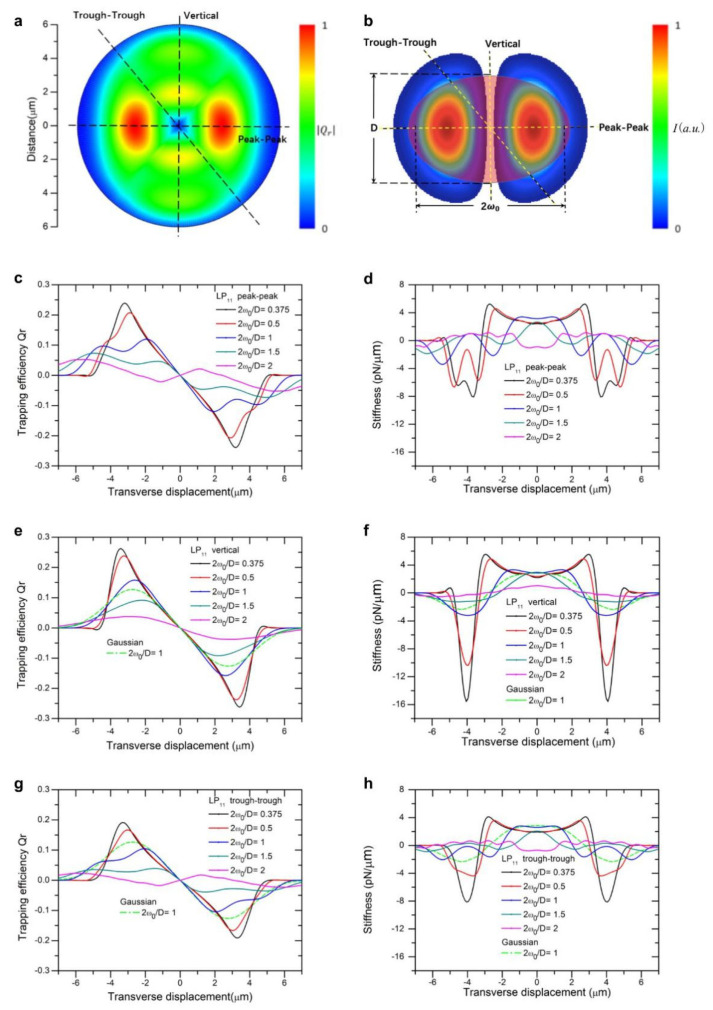
Landscape of trapping force of an LP_11_ beam. (**a**) Normalized two-dimensional (2D) trapping force distribution of LP_11_ mode; (**b**) Cross-sectional view of a spheroid cell trapped by an LP_11_ beam; (**c**) LP_11_ beam trapping efficiency vs. displacement curves along the peak-to-peak direction; (**d**) LP_11_ beam trapping stiffness vs. displacement curves along the peak-to-peak direction. (**e**) LP_11_ beam trapping efficiency vs. displacement curves along the vertical direction; (**f**) LP_11_ beam trapping stiffness vs. displacement curves along the vertical direction; (**g**) LP_11_ beam trapping efficiency vs. displacement curves along the trough-to-trough direction; (**h**) LP_11_ beam trapping stiffness vs. displacement curves along the trough-to-trough direction.

**Figure 2:**
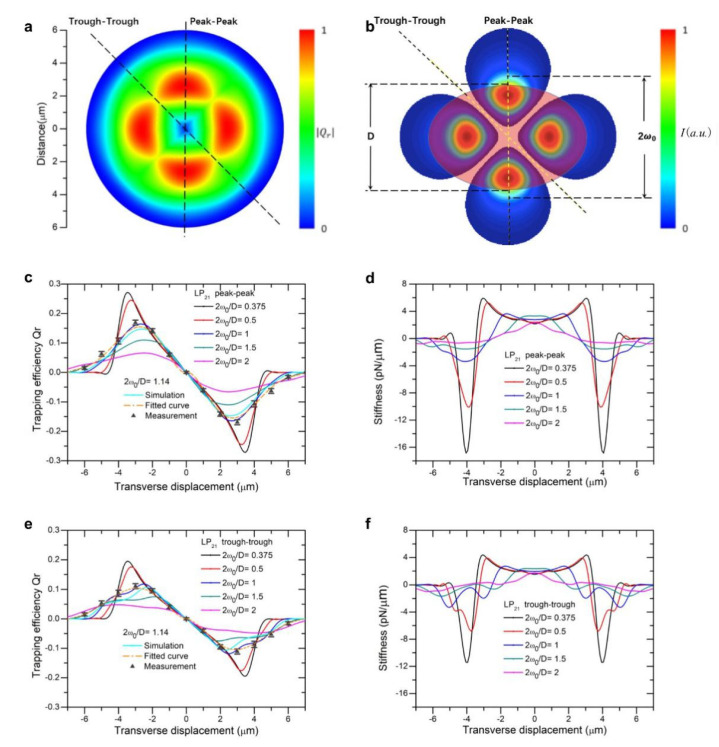
Landscape of trapping force of an LP_21_ beam. (**a**) Normalized 2D trapping force distribution of LP_21_ mode; (**b**) Cross-sectional view of a spheroid cell trapped by an LP_21_ beam; (**c**) LP_21_ beam trapping efficiency vs. displacement curves along the peak-to-peak direction; (**d**) LP_21_ beam trapping stiffness vs. displacement curves along the peak-to-peak direction; (**e**) LP_21_ beam trapping efficiency vs. displacement curves along the trough-to-trough direction; (**f**) LP_21_ beam trapping stiffness vs. displacement curves along the trough-to-trough direction.

**Figure 3:**
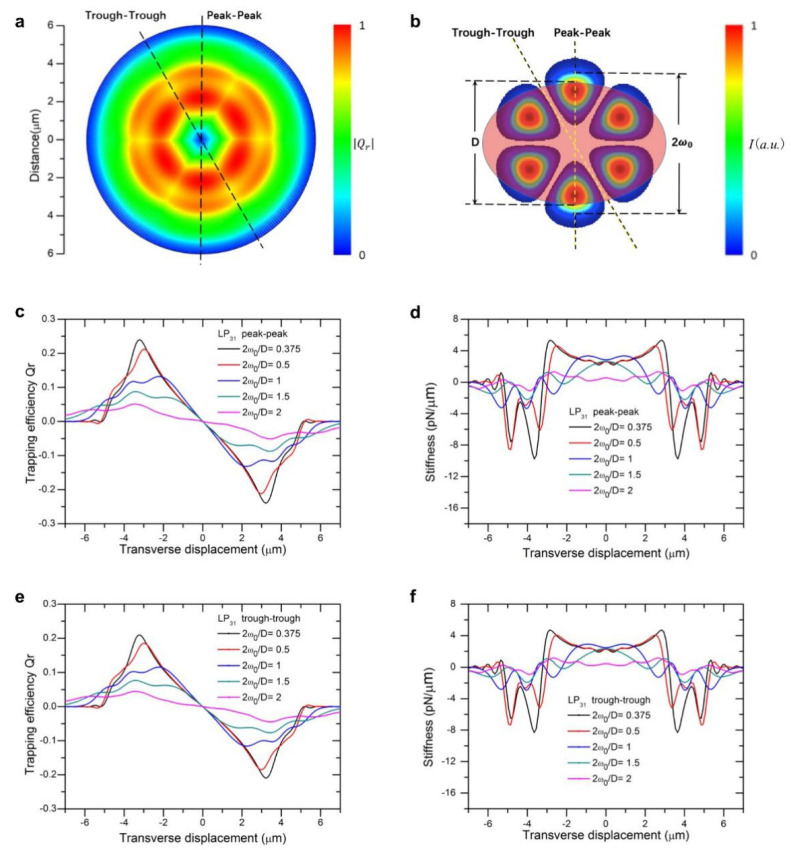
Landscape of trapping force of an LP_31_ beam. (**a**) Normalized 2D trapping force distribution of LP_31_ mode; (**b**) Cross-sectional view of a spheroid cell trapped by an LP_31_ beam; (**c**) LP_31_ beam trapping efficiency vs. displacement curves along the peak-to-peak direction; (**d**) LP_31_ beam trapping stiffness vs. displacement curves along the peak-to-peak direction; (**e**) LP_31_ beam trapping efficiency vs. displacement curves along the trough-to-trough direction; (**f**) LP_31_ beam trapping stiffness vs. displacement curves along the trough-to-trough direction.

Under the illumination of a focused beam of a high-order mode of LP_11_, LP_21_, or LP_31_, we performed computation of a two-dimensional (2D) map of the optical trapping forces acting on a spheroid bioparticle. The parameters used are the following: a spheroid yeast cell with a refractive index of n = 1.400 suspended in DI water of n = 1.330, with a short axis *D* = 4.2 μm and eccentricity ranging from 1 to 2. Figure 1a, Figure 2a and Figure 3a show the two-dimensional landscape of trapping efficiencies (absolute value) generated by beam of LP_11_, LP_21_, and LP_31_ mode, respectively (eccentricity of spheroid is set at 1.31, 2*ω*_0_/*D* = 1.14). Trapping efficiency is converted to trapping force by a multiplication factor of nP/c, with P being the beam power, c the speed of light, and n refractive index of suspension liquid. For these high-order modes, beam size 2*ω*_0_ was defined as the double distance from the beam axis where the intensity drops to 1/e^2^ (≈13.5%) of the maximum value, the same as that for a LP_01_ Gaussian beam. Laser power was set to be 10 mW in the following for stiffness calculations and wherever a light power input was necessary. 

For different ratios of beam to particle size, we plot trapping efficiencies and stiffness as a function of displacement from beam center along two directions, peak-to-peak, vertical and trough-to-trough, as indicated in Figure 1b, Figure 2b and Figure 3b using axial symmetry of beam profile overlaid with the particle. Compared with a Gaussian beam of the same size, the maxima of trapping force is close to those of modes LP_11_ (2*ω_0_/D* = 1, green dotted line in Figure 1e,f), yet the force curve exhibits ripples along the peak-to-peak direction, owing to restoring forces acting on the particle by two inline traps consecutively when the particle is displaced, as shown in Figure 1c,d. The maximum trapping force increases with decreasing ratio of beam size to particle size, as expected for high intensity beams. Along the vertical direction, peak trapping force is slightly higher than that along the peak-to-peak direction (Figure 1c verses Figure 1e), attributable to simultaneous action of two traps formed by LP_11_ mode field. But an interesting feature about these inline two traps is that trapping force in the vertical direction beyond maxima falls off much faster than that in the peak-to-peak direction, for the reason that action of trap force ceases soon after overlap reduces between particle and two traps, see Figure 1e for 2*ω_0_/D* = 1, 0.5, and 0.375. It is also noted that the calculated stiffness swings fast from positive to deeper negative peaks, shown in Figure 1f as oppose to Figure 1d. Close to the origin, trap stiffness continuously deviates from its value at the origin instead of holding constant, similar to the distribution of a focused Gaussian mode [23]. In the peak-to-peak direction, stiffness curves dictate characteristics of trapping forces exerted by two separate traps simultaneously by LP_11_ mode. When 2*ω_0_/D* increases and exceeds 1, the width of trapping force becomes wider and peak value decreases significantly, which makes the capture and transport of particles not secured. Figure 1g,h shows trapping efficiency and stiffness as a function of displacement along the trough-to-trough direction, where maxima trapping force is about 40% lower than those along the vertical direction.

For LP_21_ mode, Figure 2 displays the landscape of trapping forces and stiffness along two directions, trough-to-trough and peak-to-peak, parallel to the short axis of the particle. An LP_21_ mode beam exhibits four lobes of field concentration regions, spheroid particle along the direction of the short axis overlaps less of the light field flux as compared with that in the direction of the long axis. We note that, for small beam sizes (2*ω_0_/D* = 0.375, 0.5, and 1), stiffness fell from positive to negative values with fast slopes, as seen in Figure 2d, and reached relatively large negative peaks. This can be attributed to the reason that one of the four light spots quickly breaks away from the overlap with the scattering particle as it is displaced in the peak-to-peak direction. While in the trough-to-trough direction, the trapping force and stiffness appear the lowest value, due to the successive reduction of overlapping areas between two of traps with the particle. Choosing one of the ratios of beam-to-particle size, 2*ω_0_/D* =1.14, we experimentally validated whether the measured force curves in two directions are consistent with predictions based on T-matrix. We were able to fit the experimental data using numerical calculations based on the T-matrix method and found that data and theory are in good agreement (Figure 2c,e). 

The LP_31_ mode exhibited higher axial symmetry of trapping efficiency and stiffness as compared with the previous two modes (Figure 3a). Although *Q_r_* decreases to a certain extent in the gaps between adjacent strong electric field distributions, the gap was much smaller than the particle size, and thereby little change in trapping forces or stiffness curves were observed. Therefore, the trapping force profiles along the two directions are fairly close, apart from that the peak value in the trough-trough direction was lower than that in peak-to-peak direction (Figure 3c vs. Figure 3e). Similar patterns were observed for trapping stiffnesses, as shown in Figure 3d,f.

**Figure 4:**
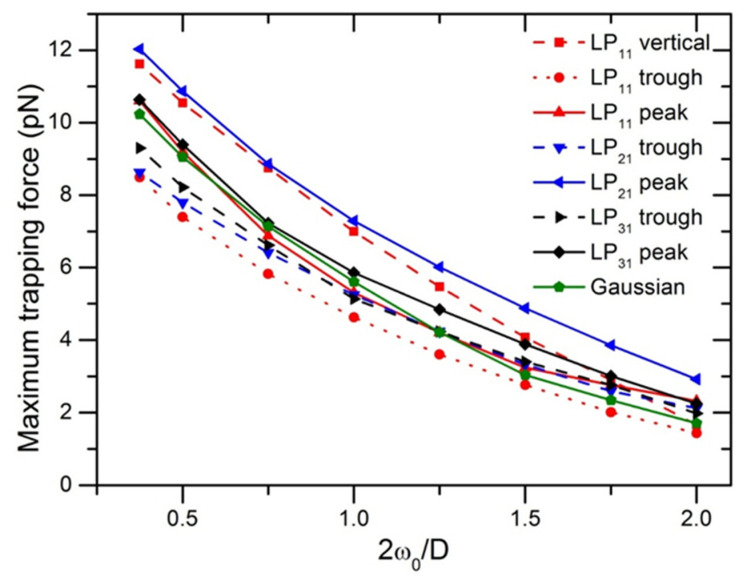
Maximum trapping forces of LP_11_, LP_21_, and LP_31_ modes along different directions as a function of ratio of beam to particle size.

Figure 4 displays the maximum trapping force in different directions as a function of ratio of beam to particle size at the illumination power of P = 10 mW. The maximum magnitudes of F_max_ from the highest to the lowest, were obtained with LP_21_, LP_11_, LP_31_, and LP_01_ Gaussian modes, respectively. For LP_11_ mode, displacing the particle along 45 directions with respect to the peak-to-peak direction, trapping force dropped about 36%, as can also be observed in the 2D force map in Figure 1a. Trapping efficiency produced by LP_11_ along the trough-to-trough direction is similar to that generated by LP_21_ along the peak-to-peak direction, but the difference from each other increases with increasing 2*ω_0_/D*, due to the two-lobed field distribution of LP_11_. The four-lobed field of LP_21_ mode surrounds a spheroid-shaped particle with a relative high stability. It is noted that F_max_ of a Gaussian beam as compared with that of other high-order modes, is in the middle of F_max_ values of other modes in different directions, as a result of axial symmetry.

### 3.2. Controlled Rotational Manipulation by LP_11_, LP_21_, and LP_31_ Modes

**Figure 5:**
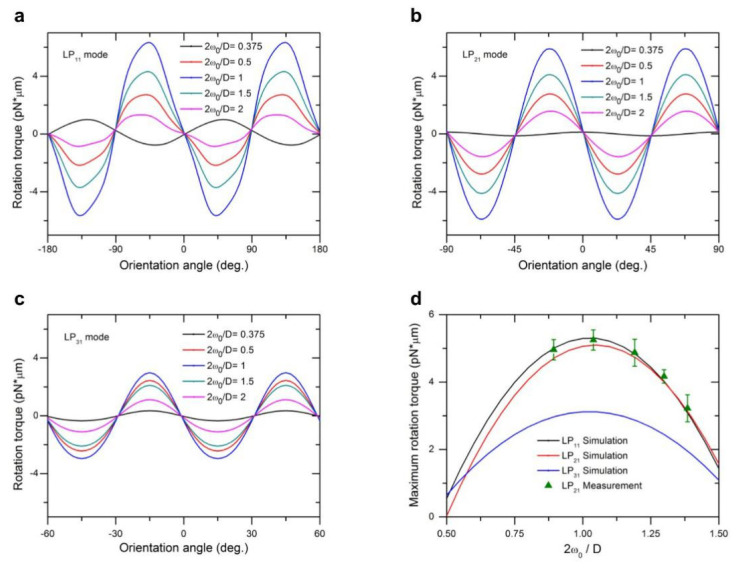
Optical torque generated by LP_11_, LP_21_, and LP_31_ modes as a function of orientation of spheroid cell with respect to radial mode field distribution. (**a**)–(**c**) For LP_11_, LP_21_, and LP_31_ mode field, respectively, rotation torques as a function of cell orientations and for different 2*ω_0_/D* ratios; (**d**) The calculated and measured maximum rotation torque as a function of 2*ω_0_/D* ratios for P = 10 mW and 2*ω_0_* = 4.8 μm.

**Figure 6:**
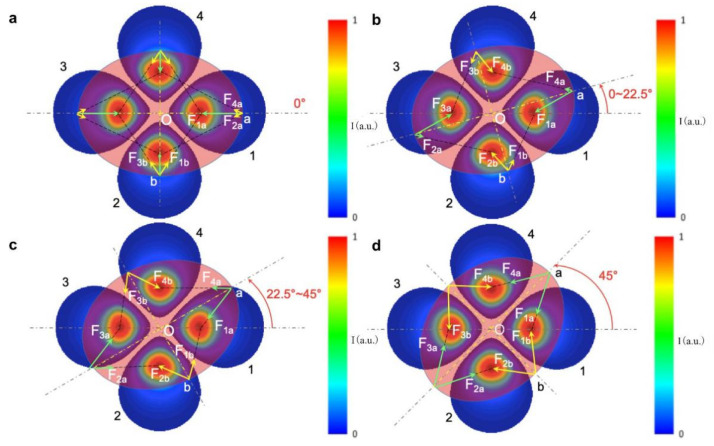
Diagrams for rotation torque analysis exemplified with an LP_21_ beam. (**a**) Rotation torque is zero due to balanced forces at the orientation angle of 0°, with respect to the symmetric axis of the mode field distribution; (**b**) A decrease in clockwise rotation torque due to reduced overlap of mode field with spheroid particle for orientation angle of 0–22.5° with respect to the symmetric axis of the mode field distribution, as indicated by sequential increase in uncovered area of beam lobe #1; (**c**) A net clockwise rotation torque generated by unbalanced force pairs at orientation angle of 22.5°–45° with respect to symmetric axis of mode field distribution; (**d**) Rotation torque returns to zero at 45°.

Utilizing the T-matrix method, we also computed optical torque *M_z_* for LP_11_, LP_21_, and LP_31_ modes, as shown in Figure 5a–c, respectively, for different ratios of beam size to particle size. Depending on the orientation of the spheroid particle with respect to the light field distribution, optical torque exhibits distinct periodic peaks of either positive or negative values, representing the restoring torque driving the particle either clockwise or counterclockwise. The optical torque *M_z_* is zero when the long or short axis of a spheroid object aligns with either the peak-to-peak or trough-to-trough directions of field distribution of a high-order mode (with the exception of LP_11_ mode, vertical direction instead of trough-to-trough direction), due to balanced pulling forces originating from multiple traps of the beam. When the orientation of the particle deviates from these symmetric directions, the optical torque will increase from zero, restoring the particle orientation back to the nearest equilibrium alignment. In Figure 6 we depict these periodic torque curves as a function of orientation angles, using LP_21_ mode as an example, where the mode field can be simplified to four centrosymmetric optical traps. At the orientation angle of 0 degrees, as shown in Figure 6a, optical forces are along symmetric axes of the spheroid particle and the net torque is zero. For an orientation of 22.5 degrees or less (Figure 6b), the LP_21_ mode field produces a clockwise restoring torque. Because beam Spots 1 and 3 are close to two endpoints of the spheroid in the long axis, each exerts a pulling force F_1a_ or F_3a_ on the spheroid, which overcomes weak forces F_2a_ and F_4a_ and forms a net clockwise driving torque. The other two light spots (Spots 2 and 4) also play a role in torsion, but the torque is small because of little change in the overlap of light field with the particle. For orientation angles of between 22.5 and 45 degrees, as indicated in Figure 6c, the overlap between beam spots with the spheroid decreases further, leading to a decrease of driving torque with increasing orientation angles. At 45 degrees, as indicated in Figure 6d, the net torque returns to zero again due to symmetric configuration. This analysis applies to the cases of LP_11_ and LP_31_ mode field as well, except the period of zero torque changes to 90 and 30 degrees, respectively. It is interesting to note, for LP_11_ mode, that the direction of the optical torque reverses for small beam sizes (2*ω_0_/D* = 0.375) due to a good match of beam size with the short axis of the spheroid particle for the purpose of rotation.

Therefore, it can be seen from Figure 5a–c that the maximum optical torques (or holding torque) occur between two adjacent symmetrical overlap configurations between the light field and spheroid particle. In Figure 5d we examine the maximum optical torque as a function of beam to particle size ratio. We also observe that the LP_11_ mode provides the maximum peak value of optical torque, while that of LP_21_ is slightly lower, and the LP_31_ mode is smaller still, retaining only 57% of the torque produced by the LP_11_ mode under the same beam power. It is clear that the holding torque decreases with an increase in the axial symmetry of the light field, approaching zero for a Gaussian beam theoretically.

Under the same illumination conditions (laser power, beam waist, and other constraints), the LP_11_ mode beam has more advantages when considering the maximum rotation torque, and the LP_31_ mode has a shorter torque cycle (backlash) of 30° as opposed to 45° of LP_21_ and 90° of LP_11_. The LP_31_ mode can realize particle rotation with a fast angular response in cyclical rotation operations. The LP_11_ mode, though its propagation is subjected to various external effects including fiber bending, twisting, or vibration, is expected to outperform other modes in cell manipulation if the beam is stabilized using a SLM and proper algorithm [18]. Overall, the LP_21_ mode provides an optical torque nearly as much as that of the LP_11_ mode, while retaining its advantages in applications requiring stable trapping and holding torque. As beam size as a function of 2*ω_0_/D* increases to match the target particle size, the scattering of angular momentum as a result of rotating light field increases and correspondingly increases maximum torque. Further increases in beam size past the target particle size leads to leakage of incoming radiation, and the torque subsequently decreases after an optimal peak. The optimal range of 2*ω_0_/D* is between 0.8 and 1.3, which offers an effective manipulation of both lateral transport and rotation of a single bioparticle with the same beam setup.

To experimentally validate peak optical torque (holding torque), a bioparticle in fluid can be rotated and maximum angular speed measured before it begins to slide instead of rotating with the light field. This is the point where the Stokes frictional torque of the liquid is equal to the holding torque. When the particle first begins rotating with a structured light field at an angular speed, the liquid-suspended spheroid-shaped bioparticle is hindered by viscous force of the suspension fluid, which is described by the following equation (also see Supplementary Materials S2):

(7) $$M_{z}-T_{z}=I\dot{\Omega },$$

where resistive torque *T_z_*, a rotational counterpart of Stokes drag force, can be expressed as:

(8) $$T_{Z}=\frac{16\pi }{3}\eta \gamma \Omega ,$$

wherein *γ* is a constant pertaining to form factors of the rotating object [26] as:

(9) $$\gamma =\frac{l_{x}^{2}+l_{y}^{2}}{l_{x}^{2}A_{x}+l_{y}^{2}A_{y}},$$

where *η* is the fluid viscosity, *I* is the moment of inertia of the bioparticle, *A_i_* and *l_i_* describe form factors and axis lengths of the spheroid particle, respectively. By solving the differential Equation (7), rotation angle *φ* can be expressed as a function of time as follows:

(10) $$\varphi \left(t\right)=c_{1}+c_{2}e^{-\frac{16\pi \eta \gamma }{3I}t}+\frac{3M_{z}}{16\pi \eta \gamma }t,$$

**Figure 7:**
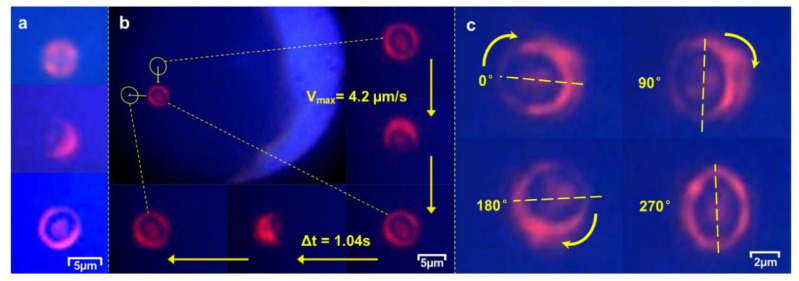
Microscopic images. (**a**) LP_21_ beam spots before and after capturing a yeast cell; (**b**) Orientation-maintained transport; (**c**) Controlled rotation of single yeast cells using an LP_21_ mode field.

Upon trapping in the focused LP_21_ beam, the yeast cell was immobilized by the four lobes of the laser beam. We further observed that a trapped cell was surrounded by a ring-like distribution of the light refracted and scattered by the cell (Figure 7a, corresponding to before and after cell capture, respectiveln be expressed as a function of time as follows: fy). Within a fraction of a second, the long axis of the asymmetrically shaped cell was observed to rotate to align itself with a lobe peak-to-peak direction (as indicated in Figure 2b) in a plane normal to the laser beam. The focused LP_21_ beam also forms a stable trap along the beam direction, levitating the captured yeast cell inside a microfluidic channel as a 3D optical trap.

The measured result, shown in Figure 5d, agrees well with that of the computed model. The experimental observations show that the holding torque of an LP_21_ mode field not only offers an efficient tool for controlled rotation of single cells and secured trapping for cell transport in different directions, but also can maintain cell orientation during transport, as displayed in Figure 7 and Supplementary Materials (Videos S1 and S2). For a laser power as low as 10 mW, the peak speed in linear transport of single cell was 4.2 μm/s and peak rotation speed was 0.54 rad/s. 

**Figure 8:**
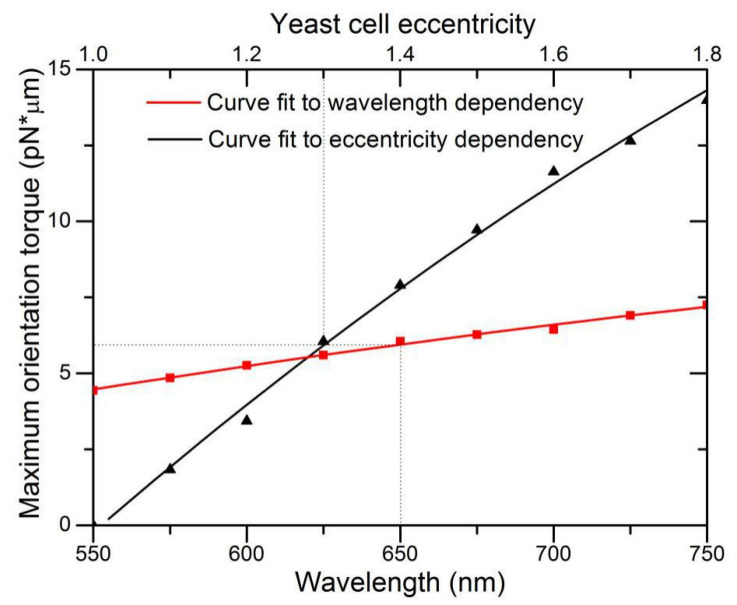
Dependence of maximum holding torque on wavelength and cell eccentricity. Dotted line denotes the case of eccentricity of 1.3 and illumination at 650 nm.

We also investigated the dependence of holding torque on wavelength and cell eccentricity. Under the illumination condition where LP_21_ mode laser power P = 10 mW and beam size 2*ω_0_* = 4.8 μm, we varied the short axis length and fixed the long axis length at 5.8 μm for the spheroid particle, and then computed maximum rotation torque as a function of the eccentricity. Figure 8 demonstrates a nearly linear increase in maximum rotation torque with eccentricity. As the short axis reduces in length, the interaction of the high-mode light field approaches the physics limit analogous to two light spots capturing a microrod at two ends, while the additional two light spots have little counteracting effect. This is also similar to the high effectiveness of rotation produced by a LP_11_ mode beam. For wavelength dependence, optical torque is theoretically linearly proportional to wavelength owing to the factor of *P/ω* to convert torque efficiency *τ_z_* to torque *M_z_*. However, the calculated result deviates slightly from linear dependence for the reason that the model T-matrix is approximated by a finite N_max_ terms of series expansion, thus, computation of all coefficients of *a_nm_, b_nm_, p_nm_*, and *q_nm_* in Equation (6) are related to the size of wavelength-related domains. 

## 4. Conclusions

We performed dynamic modeling and parameter optimization of trapping force and rotational torque of SFT using a high-order mode. First, a general dynamic model of the trapped cell, taking into account cell rotational motion, is established for controlled motions of single cells using one of the high-order modes of SFTs. Second, the relationship between the driving torques to the cell and different fiber modes is characterized. Third, the optimal beam sizes of the structured light field were obtained, applicable to complex manipulation operations of SFTs with either single-core or multiple-core fibers. Using a T-matrix method-based scattering field simulation, a two-dimensional trapping force distribution, as well as optical torques are obtained for high-order LP_11_, LP_21_, and LP_31_ mode SFT beams, under a number of illumination conditions. Both the trapping force distribution and optical torques were analyzed in detail for the optical manipulation of single cells. The results show that both LP_11_ and LP_21_ mode have a moderate high trapping force and large torque under the same illumination conditions, while LP_31_ has better control of cyclical particle rotation due to small angular spacing, and thereby backlash between adjacent zero torque orientations. The optimal range of the ratio of beam-to-target-cell size was obtained to be between 0.8 and 1.3, for the effective manipulation of both transport and controlled rotation of a single cell simultaneously. An optimized high-order mode single-fiber optical tweezer demonstrates the potential to be implemented with inexpensive optical components, easily integrated with other micro-optical devices, and enables unprecedented capability in minimal impact complex manipulations to a range of biological research and medical applications.

## Data Availability

Not applicable.

## Supplementary Materials

The following are available online at https://www.mdpi.com/2072-666X/12/2/226/s1, S1: Derivation of expression for LPlm mode in cylindrical coordinates; S2: Derivation of Stokes viscous resistance torque for a sphe-roid-shaped single cell; Video S1: Orientation-maintained translations of single cell, Video S2: Controlled rotation of single yeast cell.
