# Supplementary material for: High-Order Fiber Mode Beam Parameter Optimization for Transport and Rotation of Single Cells
Source: Micromachines (Basel). 2021 Feb 23;12(2):226. doi: 10.3390/mi12020226 (PMC7926556; doi:10.3390/mi12020226)
Supplement: Supplementary file 1 [file micromachines-12-00226-s001.zip › micromachines-1117478 supplementary/supplement materials/supplementary materials S1.docx]

High-order fiber mode beam parameter optimization for transport and rotation of single cells: supplementary materials

**S1: Derivation of expression for LPlm mode in cylindrical coordinates**

Starting from Helmholtz equation for z-component in Cartesian coordinates:

(S1)

We rewrite the equations in cylindrical coordinates：

(S2)

(S3)

(S4)

(S5)

(S6)

(S7)

Where is normalized transverse phase parameter, is normalized transverse attenuation parameter, and is normalized frequency, with . By introducing normalized phase constant b=W2/V2, then U and W take the form ， .

By imposing boundary condition that the tangential component of E-field and magnetic field Hz1 is continuous on the boundary of Ra=1, we have:

(S8)

(S9)

In fiber core, the electromagnetic field is given by

(S10)

And inside the cladding of a fiber, electromagnetic field is expressed as:

(S11)

While the field strength on the interface between fiber cladding and core is linked by the following boundary conditions：

(S12)

By inputting the above EM field components into the first two boundary conditions, we have:

(S13)

By multiplying the two equations, then

(S14)

and using β2=ω2μ0ε0:

(S15)

For weak-bounding fiber n1/n2 →1, therefore a characteristic equation is obtained:

(S16)

When l=0, there exist only TE and TM modes. When l≠0，there exist both EH and HE modes, which are consisted of both EZ and HZ components. The plus sign in the characteristic equation is for EH modes, while the negatice sign is for HE modes. i.e.

For TE and TM modes coexisting：

(S17)

For EH modes：

(S18)

For HE modes：

(S19)

the latter two equations can be simplified to be：

EH modes：

(S20)

HE modes：

(S21)

LPlm modes is a linear superposition of HEl+1,m and EHl-1,m modes, with its characteristic equation of

(S22)

On fiber end face, the radiation field of Fraunhoff region is given by:

(S23)

Where

(S24)

(S25)

After solving for EFF, we obtained the following expression:

(S26)

For LPlm mode：

(S27)

References

1. Okamoto K. Fundamentals of optical waveguides, Elsevier Inc. (2006) ISBN 13: 978-0-12-525096-2
2. C. L. Chen, Foundations for Guided-Wave Optics, Wiley-Interscience, New York (2006) ISBN-13: 978-0471756873.
