# Supplementary material for: High-Order Fiber Mode Beam Parameter Optimization for Transport and Rotation of Single Cells
Source: Micromachines (Basel). 2021 Feb 23;12(2):226. doi: 10.3390/mi12020226 (PMC7926556; doi:10.3390/mi12020226)
Supplement: Supplementary file 1 [file micromachines-12-00226-s001.zip › micromachines-1117478 supplementary/supplement materials/supplementary materials S2.docx]

High-order fiber mode beam parameter optimization for transport and rotation of single cells: supplementary materials

**S2. Derivation of Stokes viscous resistance torque for a spheroid-shaped single cell**

Starting from Navier-Stokes equation:

$\frac{\partial\overrightarrow{v}}{\partial t}+(\overrightarrow{v}\cdot\nabla)\overrightarrow{v}=-\frac{1}{\rho}\nabla P+\frac{\eta}{\rho}\nabla^{2}\overrightarrow{v}$ (S1)

For steady-state flow：

$\frac{\partial\overrightarrow{v}}{\partial t}=0$ (S2)

And for small Reynolds number, we can omit the term：$(\overrightarrow{v}\cdot\nabla)\overrightarrow{v}\approx0$ (S3)

Thus the dynamic equation of steady flow of incompressible fluid with small Reynolds number is：

$\nabla P=\eta\nabla^{2}\overrightarrow{v}$ (S4)

Morphological characteristics of ellipsoids：

$\left\{\begin{matrix} A_{\text{i}}=\int_{0}^{\infty} \frac{ds}{(s+l_{i})\sqrt{(s+a^{2})(s+b^{2})(s+c^{2})}} \\ A=\int_{0}^{\infty} \frac{ds}{\sqrt{(s+a^{2})(s+b^{2})(s+c^{2})}} \end{matrix}\right.$ (S5)

Where a,b and c are the axis length of the three components of the ellipsoid along x, y and z directions.

So the viscous force of an ellipsoid in a steady flow fluid is

$F_{i}=\frac{16\pi\eta}{A+l_{i}^{2}A_{i}}v_{i}$ (S6)

The Stokes resistance torque exerted on the ellipsoid rotating along Z coordinate axis is：

$T_{\text{z}}=\frac{16\pi}{3}\eta\frac{l_{x}^{2}+l_{y}^{2}}{l_{x}^{2}A_{x}+l_{y}^{2}A_{y}}\theta^{\cdot}$ (S7)

References

1. inter W. T. and Welland M. E. , Dielectrophoresis of non-spherical particles. Journal of Physics D Applied Physics, 2009, 42(4):045501.
